# The meaning of the recovery process and its stages for people attending a mental health day hospital: A qualitative study

**DOI:** 10.1111/hex.13965

**Published:** 2024-01-07

**Authors:** Ana Ventosa‐Ruiz, Antonio R. Moreno‐Poyato, Teresa Lluch‐Canut, Isabel Feria‐Raposo, Montserrat Puig‐Llobet

**Affiliations:** ^1^ Department of Public Health, Mental Health and Maternal and Child Health Nursing Nursing School, Universitat de Barcelona L'Hospitalet de Llobregat Spain; ^2^ Benito Menni Centro de Atención a la Salud Mental, Sant Boi de Llobregat Barcelona Spain; ^3^ Nursearch Grup de Recerca en Cures Infermeres de Salut Mental, Psicosocials i de Complexitat Barcelona Spain

**Keywords:** day hospital, mental health, nursing, qualitative study, recovery process

## Abstract

**Introduction:**

This study sought to explore the meaning of the recovery process and its stages from the perspective of people attending a mental health day hospital.

**Methods:**

A descriptive exploratory qualitative study was carried out. Semi‐structured interviews were conducted with people attending a mental health day hospital. The data were analysed deductively by means of content analysis.

**Results:**

The participants described the recovery process as a process based on three pillars; the attitude towards recovery, hardship, and the effort required throughout the process. Regarding the stages of recovery, for the participants in the first stage of the process (Moratorium), the search for hope was the most important element. In the second stage (Awareness), the reestablishment of their identity, through the acceptance of the consequences derived from the mental health problem, together with being able to feel full and fulfilled, were the most outstanding elements. In the third stage (Preparation), participants highlighted the search for meaning in life, facing their fears and the process with an open mind. Finally, the last two stages (Rebuilding and Growth) were related to taking responsibility and empowerment for recovery.

**Conclusions:**

The results of this study provide insight into the perception of the recovery process and its stages in people attending a mental health day hospital. These findings may contribute to aligning the nurse–patient perspective, helping nurses to understand the key elements of patients according to their stage of recovery, and thus be able to subsequently individualise interventions.

**Patient and Public Contribution:**

This study was based on interviews with 15 patients receiving treatment at an adult mental health day hospital. This study would not have been possible without their participation.

## 1 INTRODUCTION

Figures on global mental health, according to the World Health Organization (WHO), in its latest report^1^ estimate that one billion people (more than one in eight adults and adolescents) worldwide suffer from a mental health problem. Moreover, these people die 10–20 years earlier than healthy people and generate an avoidable economic burden.

In recent years, the model of mental health care has changed from a rehabilitative model, with the focus on the professional, to a recovery model, where the main protagonist is the person suffering from a mental health problem.^2^ The WHO guidelines support the recovery model,^3^ recommending the integration of a restorative vision into mental health care policies. In this context, person‐centred care acquires great relevance.^4^ Mental health nurses must have an in‐depth understanding of the perspective of people with mental health problems on what recovery means to them throughout the various stages of the recovery process and their level of care.

### 1.1 Background

In recent decades, different authors have contributed to the study of the phenomenon of the recovery process in the field of mental health. In the 1980s, Deegan^5^ broke away from previous models of mental health care and defined the recovery process as ‘the lived or actual life experience of people as they accept and overcome the challenge of disability, so that they experience a new sense of self within and beyond the limits of disability’. Subsequently, Anthony^6^ outlined the basis of the recovery model, defining it as ‘a way of living a satisfying life, full of hope and contribution, even within the limitations caused by the disease’. Later, Andresen et al.^7^, ^8^ also studied the phenomenon from the perspective of the processes a person goes through in their recovery process. In their work, they described four component processes and five stages that the person undergoes during the recovery process. The stages identified were: (i) Moratorium: A time of withdrawal characterised by a deep sense of loss and hopelessness, (ii) Awareness: Realising that all is not lost and that a full life is possible, (iii) Preparation: Weighing strengths and weaknesses in relation to recovery and beginning to work on developing recovery skills, (iv) Rebuilding: Actively working towards a positive identity, setting meaningful goals and taking control of one's life and (v) Growth: Living a full and meaningful life, characterised by self‐management of illness, resilience and a positive sense of self. These stages are sequential, and therefore, the last stage, growth, constitutes the result of the recovery process.^8^ On the road to recovery, and independently of the stage the person is in, for Andresen et al.^8^ people experience different psychological states which were termed component processes. These four processes are; (i) finding and maintaining hope—believing in oneself, having a sense of self‐control and optimism for the future; (ii) re‐establishment of a positive identity—finding a new identity that incorporates the illness but maintains a positive sense of self; (iii) finding meaning in life—understanding the illness; finding meaning in life despite the illness; dedicating oneself to living; (iv) taking responsibility for one's life—feeling in control of the illness and in control of one's life. For Andresen et al.,^8^ given the highly personal sense of recovery, the model should be considered flexible in terms of the order and means by which the person moves through the processes.

More recently, Leamy et al.^9^ developed the CHIME model based on a systematic review,^9^ using an acronym that represents the five essential elements of recovery in mental health: Control, Hope, Identity, Meaning and Empowerment. For Leamy et al.,^9^ control refers to the person's ability to have control over one's life and treatment, including the ability to make informed decisions and to manage symptoms. Hope is the belief and confidence in the possibility of improvement and recovery. Identity illustrates the importance of maintaining and developing a positive identity and healthy self‐esteem. Meaning appeals to the importance of finding purpose and meaning in life, including participation in meaningful activities and community. Finally, empowerment is the ability of individuals to gain the necessary control and influence to improve their well‐being and achieve their goals. The CHIME model is based on the idea that recovery in mental health is not only about managing symptoms but also about promoting the well‐being and long‐term development of the individual. This model is used in mental health care as an individual‐centred approach and emphasises the importance of working with each person to develop a personalised treatment plan that addresses these five essential elements of recovery.^9^

In any case, different models of recovery have been questioned for their dualism and lack of depth in understanding the complexity inherent to mental health problems. Such models oversimplify recovery processes, ignoring the many individual and contextual variables that influence each person's experience of mental health problems.^10^ Different models of recovery have suggested the need to align the concept of recovery between people with mental health problems and professionals, as differences in understanding can lead to frustration and abandonment of the process,^8^, ^9^ understanding that there are profound implications in the sensitivity and appropriateness of therapeutic communication.^11^ Some studies suggest that health professionals' conception of the concept of recovery in users of mental health services is strongly linked to the concept of medical recovery.^12^, ^13^ However, recovery according to people receiving mental health care is understood as an ongoing quest in life, underpinned by five thematic pillars: ‘finding meaning’, ‘an invisible disability’, ‘empowerment and agency’, ‘connection’ and ‘the passage of time’.^14^ Specifically, in studies of people diagnosed with schizophrenia, recovery was perceived not only as the absence of symptoms, rather it was found to be important to be able to work, regain functioning, have adequate emotional stability, and be free of medication.^15^ Similarly, in people diagnosed with bipolar disorder, recovery was identified with productivity, engagement, social participation and symptom reduction.^16^

From a sociocultural perspective, there are differences in the formulation of the concept of recovery between developed countries and other low/middle‐income countries.^17^ For the latter, studies frame recovery as a personal journey that occurs along a continuum, where social relationships are emphasised as facilitators of recovery and spirituality is a facilitator and an indicator of recovery. These differences in how recovery is understood reflect the importance of framing the concept of personal recovery in relation to the local needs and contextual issues of each territory.^17^ Likewise, many social factors are known to be related to recovery, where the consequences of illness, financial precariousness and caregiver burden can put a strain on essential social needs, representing barriers to recovery.^18^

In relation to the area of care, the evidence points to certain nuances in the experience and meaning of the recovery process on behalf of people with mental health problems. People cared for in the community give special relevance to interpersonal and self‐connection in their recovery process, through inclusion in one's own community, receiving support when needed and being able to progress through loss.^19^ However, in other more restrictive areas, such as forensic psychiatric nursing, the salient aspects of the recovery process for people with mental health problems were disconnection, hopelessness, negative identity experience as an offender, lack of meaning and perceived disempowerment.^20^, ^21^, ^22^

In the context of mental health care in day hospitals, mental health care is offered within the community at times when the person's needs are acute. This care is provided by a multidisciplinary team for a period of no more than 3 months and avoids admission to an acute care unit, which is usually considered detrimental to the lives of people with mental health problems.^23^

The importance of aligning the perspectives and expectations between nurses and people with mental health problems^24^ is evident for clinical practice, not only regarding the meaning of the recovery process but also the knowledge of its stages.

Knowledge gaps have been previously identified in terms of mental health service users' conception of the recovery process.^9^ Specifically, no studies have been found which explore this process in relation to the stage of care of people who are treated in mental health day hospitals.

Considering that there is hardly any evidence that provides knowledge about this phenomenon in specific care contexts such as mental health day hospital care, it is necessary to further explore this matter from the perspective of people with mental health problems and the meaning they assign to the recovery process and its stages.

## 2 METHODS

### 2.1 Aims

This study sought to explore the meaning of the recovery process and its stages from the perspective of people treated in adult mental health day hospitals.

### 2.2 Design

A descriptive‐exploratory qualitative study was carried out. This approach was used because it enables us to examine reality and not simply observe it, thus it was identified as the most suitable method for the purpose of the study.^25^ This study was guided by the consolidated criteria for reporting qualitative research checklist (COREQ 32).^26^

### 2.3 Sample/participants

The study participants were people with mental health problems who were attended to at an adult mental health day hospital. The inclusion criteria were being over 18 years of age at the time of admission, accepting the conditions of the study and signing the informed consent form. Individuals who had been attended for less than 1 week or whose physical or psychological conditions did not enable them to participate in the study were excluded. However, there were no participants who met these conditions during recruitment. A purposive sampling by maximum variation was carried out considering pre‐established profiles. None of those invited to participate in the study refused to do so.

### 2.4 Data collection

Data were collected through semi‐structured interviews conducted by the principal investigator of the study, a female mental health nurse specialist. These interviews were conducted at the time of the persons' admission to an adult mental health day hospital, and therefore the interviewer had no prior relationship with the interviewees. The document by Kallio et al.^27^ was used to define an interview script, after a pilot test was carried out to verify its suitability. This pilot interview was conducted with a person with mental health problems who collaborates with the unit in which the study was conducted. After the pilot test, the usefulness of the questionnaire was corroborated, and no changes were made. The questions included in this script were: What do you understand by recovery? What importance do you attach to this process? How would you describe your recovery process? What is your goal that you need to achieve to feel recovered? What can help you to improve and what would not? The interviews were conducted in the mother tongue of the interviewees to eliminate language barriers by the nurse on duty. The interviews took place in a comfortable environment with limited interruptions at the facilities of the day hospital. Only the researcher and the participant were present during the interview. The average duration of the interviews was 25 min. The interviews were recorded, with the consent of the interviewees, using a voice recorder after informing the participants of the objectives of the study. No repeat interviews were necessary. Participants were given the possibility of interrupting the interview at any time without having to give any explanation. The interviews were subsequently transcribed by a professional transcription company, and in a third step the participants confirmed that the content of the transcriptions was correct. In this third step, none of the participants made any corrections to the proposed transcript. To complement the information, field notes were taken after the interview was conducted. Data collection took place between September and December 2021. The selection of participants took place until the a priori thematic saturation was reached^28^ based on the stages of the recovery process of the Andresen model.

### 2.5 Ethical considerations

This study was approved by the Ethics Committee of the institution where the research was carried out, FIDMAG Hermanas Hospitalarias (PR‐2020‐10). To participate in the study, the subjects had to read and accept the informed consent form. They were also offered the possibility of withdrawing from the study at any time without the need to state a reason. Participants did not receive financial compensation for their participation. To ensure anonymity, each participant was assigned an alphanumeric participant code. Also, the stored data were protected by encryption and password protection. During recruitment, participants were informed of their voluntary participation in the study, explaining that their refusal to participate would have no consequences on the quality of their treatment, and that all the information derived from their participation was confidential and would not have any effect on their treatment.

### 2.6 Data analysis

A qualitative content analysis was performed.^29^ The information derived from the interviews was transcribed verbatim, and in a second step, validated by the participants to verify authenticity. The texts were divided into descriptive codes according to their semantic content. These codes were grouped into analytical subcategories to group the initial codes according to linguistic units. Finally, considering the semantic analysis of the above subcategories, they were grouped into categories deductively according to the stages of the retrieval process of the Andresen model.^8^ These stages were mainly carried out by the first author and were continuously and critically discussed and reflected upon within the research team. The Nvivo 12 software was used for this categorisation.

### 2.7 Rigour

To ensure the validity and rigour of the present study, strategies suggested by Lincon and Guba.^30^ were used based on credibility, transferability and confirmability. The transcriptions of the recorded interviews with subsequent confirmation by the interviewee ensure the veracity of the data obtained and credibility. To ensure confirmability, a field diary was kept by the main researcher to consider the researcher's impressions and emotions, which also helps to achieve neutrality in keeping the experiences far from any possible biases. Concerning transferability, the sampling process and the characteristics of the sample have been scrupulously described.^31^ In terms of reflexivity, the author comes from a health care background and has experience in mental health research, as well as experience in conducting qualitative interviews. The interviewer established a therapeutic bond with the participants and endeavoured to be neutral throughout the interview, avoiding bias and facilitating the free expression of opinions and positions.^32^

## 3 FINDINGS

Fifteen people participated in the study. Of these, 73% were women (*n* = 11) and 27% were men (*n* = 4). The mean age was 45 years (min 20–max 58). Regarding the diagnosis, 40% of the participants had an affective disorder (*n* = 6), 27% had an anxiety disorder (*n* = 4), 13% had a psychotic disorder (*n* = 3) and 13% had a personality disorder (*n* = 2). The mean number of years since the diagnosis of the mental health problem was 8.8 years (min 1–max 21). The employment status of participants was as follows: 33% (*n* = 5) were granted temporary incapacity, 13% (*n* = 2) were students, 20% (*n* = 3) were unemployed, and 33% (*n* = 5) were retired. Concerning the country of birth, 73% (*n* = 11) were of Spanish nationality, and 27% (*n* = 4) were of foreign origin.

**Figure 1:**
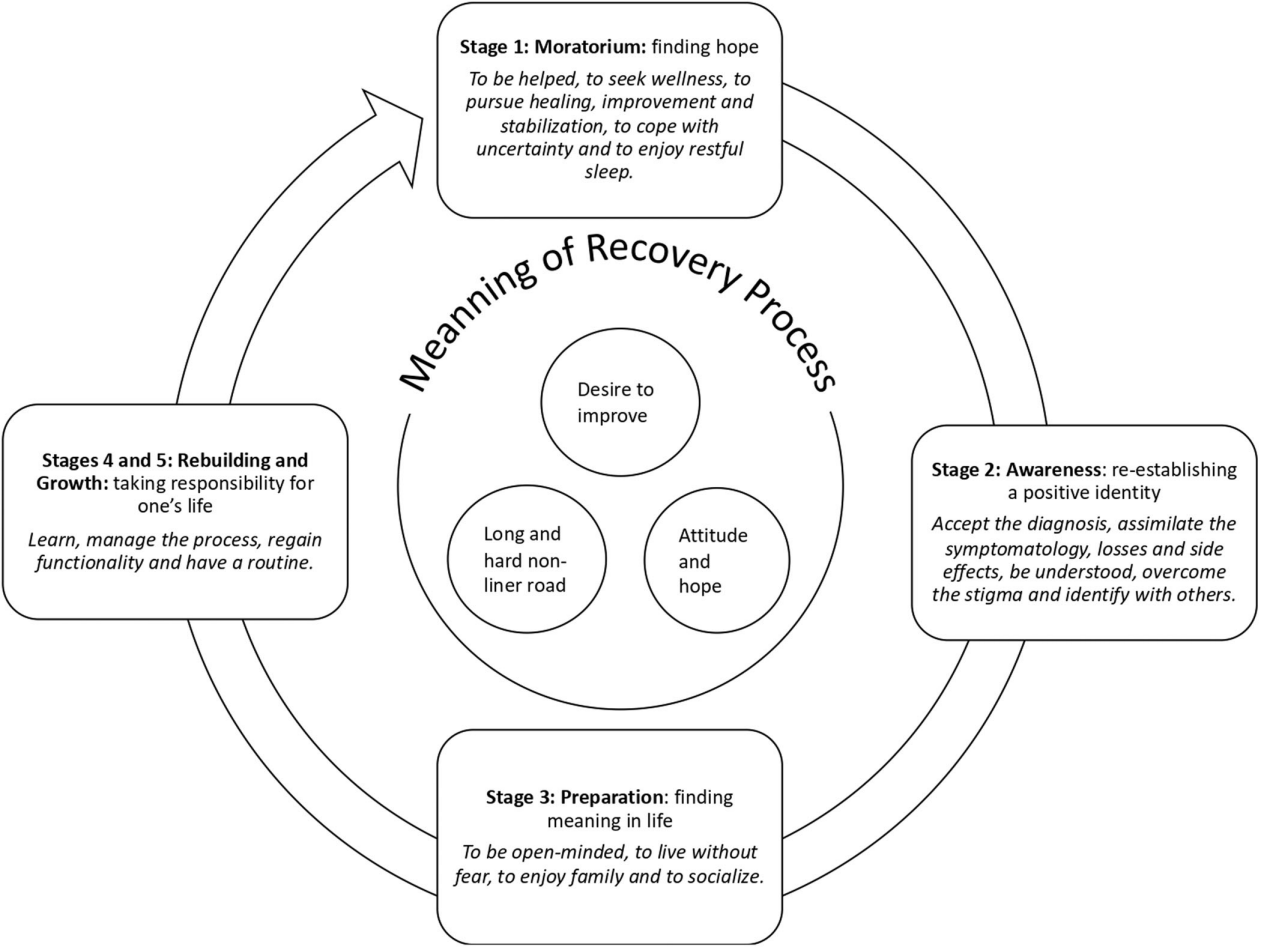
The meaning of the recovery process and its stages for people attending a mental health day hospital.

As a result of the data analysis, a total of 378 minimum units of meaning were identified. After categorising these by meaning, they were grouped into a total of 29 categories, of which seven corresponded to the definition of the process as such and 22 to the different stages of the recovery process, which were finally grouped into four main themes. These themes corresponded to the stages of the recovery process according to Andresen et al.'s^8^ model except in the case of rebuilding and growth which were agglutinated into a single theme (Figure 1).

### 3.1 The meaning of the recovery process

For the participants, the meaning of the recovery process was based on three pillars; the attitude towards recovery, hardship and the effort required during the process. In this regard, the recovery process represented a path based on the desire to improve. ‘Recovery would mean to be better or completely well, compared to how you are at the beginning of your recovery’ (p. 5). Consequently, this process was highly dependent on the attitude towards recovery, which played a decisive role. ‘At the moment I give it a lot of importance to it, because I want to recover as much as possible and I have a goal, so I want to fight for it and be able to live again’ (p. 10).

In this context, people with a mental health problem also emphasised the rawness and harshness of this process. ‘Because you have to do your part, it is very hard, very hard, but you have to try and you have to fight, because it's the only way to do it’ (p. 9). In addition, participants verbalised the importance of themselves in this process, as they defended that recovery does not depend on professionals but rather on the person affected by a mental health problem. ‘Here you get 20 percent and 80 percent depends on yourself’ (p. 4).

### 3.2 Moratorium: Seeking hope

For the participants, hope was seen as a first step in their recovery process. This included aspects related to the recovery of personal well‐being, linked to the reestablishment of certain aspects of their life before the onset of the mental health problem. ‘To regain my values, my wellbeing, my objectives, my relationship with others, in other words, to have a life, to try to have a life that is as healthy, fulfilling and satisfying as possible’ (p. 10). Notably, receiving support from others was a major aspect for them. ‘Family and professionals push you to do it, and when you say “I can't do it anymore”, they say “no, come on, you can do it”’ (p. 8). In addition, one aspect highlighted as a facilitating element at this stage of the process was getting enough rest, emphasising the importance of sleep, in terms of quality and quantity. ‘To regain the feeling of being sleepy and say “I'm so sleepy, I'm going to bed”, to sleep and wake up rested, I lost that many years ago, I just sleep because I take medication to sleep, otherwise I wouldn't sleep’ (p. 3). Conversely, at this stage of recovery, for the participants, the presence of uncertainty was discovered as an element that conditioned their ability to move forward in the process. ‘It's not the same when it happens to you the first time, and you don't know what's happening to you, then when it happens to you and you know that what's happening to you is normal, that it's happening to other people’ (p. 12). In fact, those people who were very early in the recovery process thought of the process as an absolute healing process. ‘For me, to recover from something is, for example, to be cured of an illness’ (p. 3).

### 3.3 Awareness: Re‐establishing an identity

Participants stated that at this stage it was essential for there to be an acceptance of the consequences of the mental health problem: *‘*to be able to accept and live my states of loneliness with tranquillity and peace’ (p. 10). Together with this acceptance, they also considered the fact of feeling fulfilled despite the symptomatology derived from the mental health problem. ‘The disease is there, and it is a disease that cannot be cured, but I do know that it is possible to live with it’ (p. 10).

At this stage of the recovery process, the people participating in the study considered the importance of feeling understood by their close environment and by professionals. ‘It has helped me to feel the understanding of my family’ (p. 14). ‘I have felt a very profound understanding on behalf of the professionals’ (p. 2).

Being able to overcome the stigma attached to mental health pathologies was a difficult but achievable goal for those interviewed ‘even though there are some prejudices about people with problems, if you deal with it in a normal way, you almost forget about it’ (p. 13).

Similarly, for the participants, identifying other people with similar problems allowed them to move forward in the recovery process. ‘It's not the same when it happens to you the first time, when you don't know what's happening to you, as when it happens to you and you know that what's happening to you is normal, that it happens to other people, what happens is that it happens to you in somehow in a different way’ (p. 12). As well as becoming aware of the losses caused by the disease. ‘I lost my job, I lost my salary, I lost everything, so I feel guilty. I have lost a lot of things’ (p. 14).

However, for the participants, the side effects of the pharmacological plan, as well as the repeated changes in the plan, were an element that made it difficult to face this stage of recovery. ‘I feel reluctance towards the pharmacological aspect, at the beginning I didn't want to take so much medication, and every time I have been taken off the medication, I have felt relieved, and every time they have increased it, again I feel down. I see it as a setback, and it doesn't have to be like that’ (p. 7).

### 3.4 Preparation: Seeking meaning in life

For the participants, in this third stage, it was essential to be able to face the process with an open mind. ‘If you open your mind to let yourself be helped, collaborate and use the tools you are given, you will recover; but if you are in denial, everything is a no, and you close your mind, it will be difficult to get out of here’ (p. 4). To this end, participants verbalised the importance of overcoming fears. *‘*My goal to achieve in order to feel recovered? Well, to effectively recover many of the things I have lost, to be able to go out, have fun, have enthusiasm for things, and overcome my fears’ (p. 3).

In this phase of the process the participants acknowledged that the family and social network is fundamental. The presence and support of the family was key, understanding that their condition has direct repercussions on the people close to them. ‘If I am not well, neither is my family’ (p. 14).

Likewise, socialisation became important as a facilitating element of the phase in which people search for a new meaning in life. ‘here I have relearned how to talk to people, because I wasn't talking to anyone’ (p. 8). ‘I've had several friends here who I think understand me and I understand them, because, although each of us has a different disease we understand when the other one isn't well, and that hasn't happened to me anywhere, of course, because nobody understood me; when I've had friends they always said, “we're tired of helping you”’ (p. 8).

In addition, the participants stated that this socialisation depended on themselves. ‘Well, during the time that I've been here I've started… Well, the most important thing is to get out of the house, and I've managed to do that thanks to coming here, by coming to the day hospital. And I have to make an effort to socialise, even though I am given the tools here; but hey, we are getting there’ (p. 4).

### 3.5 Rebuilding and growth: Taking responsibility for recovery

Regarding these last stages of the recovery process, the people interviewed clearly felt that the process depends largely on oneself, emphasising both the responsibility they have in the process and the importance of their attitude. ‘It mainly depends on oneself. Of course, help is always appreciated’ (p. 9).

They also emphasised the need to learn about themselves and their disease when they were at this stage ‘My goal is to learn how to cope with the process’ (p. 11). ‘it is very important to learn how to improve’ (p. 11).

Another central focus of the significance of the recovery process was functionality, as a key element of the recovery process. ‘being responsible for myself, doing things that I can't do’ (p. 14).

Finally, for those interviewed, the need to have a daily routine was important to take responsibility for their own lives. ‘now I have the obligation to get up every day, to shower, to get dressed, to come here, to try to make the most of the hours that I'm here’ (p. 3).

## 4 DISCUSSION

The study aimed to shed light on the meaning of the recovery process and its stages for people attended at an adult mental health day hospitals. The results show that, in relation to the recovery process, the participants agreed that it is a process based on the desire to improve, in which their attitude is key. Thus, recovery is a hard and costly process, which depends on oneself. These findings coincide with those of Kidd et al.^14^ based on a participatory action research conducted in a mental health service, involving caregivers, clinicians and people with mental health problems, which illustrated the meaning of the process of recovery with the metaphor ‘recovery as an ongoing quest in life’. Along these lines, a study by Leamy et al.^9^ developed a theoretical framework about the recovery process, concluding that it is a personal, active and difficult process. The similarities between the meaning of this process and the Prochaska and DiClemente^33^ cycle are striking, as both processes are based on the desire to improve and the motivation to change. However, in the Prochaska and DiClemente cycle a behavioural change is pursued, and in the recovery model more ambitious objectives are covered, which bring more aspects into play and not only a behavioural improvement. According to our study findings, the participants' narratives followed the stages proposed in Andresen et al.'s^7^ model despite the well‐known simplification of the recovery processes in some models.^10^

The findings indicate that in the first stage of the recovery process, the most important aspect for the participants was the search for hope. As in other studies, for people treated in day hospitals, returning to how they were before the onset of a mental health problem was a source of hope.^14^, ^34^, ^35^ Nonetheless, for people with mental health problems, the support received from their environment was the most important element in this stage of the process.^36^, ^37^ This included family support but also professional support.^34^ Another important finding at this stage of the recovery process was the negative experience of uncertainty regarding the problem and the future. This has been found to be relevant and essential in other research, which focuses on the general population's lack of knowledge of mental health problems^34^, ^36^ At this stage of the recovery process, increased mental health literacy would help those affected to be more confident and able to adopt a more positive outlook on life with a greater degree of empowerment.^9^, ^38^ In fact, for people with mental health problems, regaining basic aspects such as sleep quality was especially important at this stage.

In the second stage of the recovery process, which is characterised by awareness,^8^ the fundamental component was to try to re‐establish their identity.^8^, ^9^ To this end, the respondents attached importance to the acceptance of the consequences derived from the mental health problems, in contrast to the previous stage in which recovery was perceived as a complete recovery.^39^ The understanding of the aforementioned difficulties by the family and professionals as a facilitator of the recovery process has been widely described in several studies.^13^, ^34^, ^40^ This acceptance on behalf of the health professionals is especially important in the case of nurses, since they are an essential component of the nurse–patient therapeutic relationship.^39^, ^41^ At this stage of the recovery process, besides the family and professionals, the importance of support and understanding from peers was also highlighted.^42^ Several studies have shown this type of support to be a cornerstone of the recovery process.^9^, ^14^, ^36^ During this period, the effects of medication and stigma stand out as hindering elements for the recovery process. Thus, stigma represents a great burden during the recovery process, encompassing both third party stigma and self‐stigma.^43^, ^44^

In the preparation stage, the participants emphasised the search for meaning in their lives. To do so, open‐mindedness and facing fears were essential. Once the participants had accepted the presence of the mental health problem and felt understood by their family, it was time for them to face new realities, therefore, they needed to be more open‐minded than before.^10^, ^34^, ^36^ Indeed, there is evidence that relates the meaning of life and the expression of anger at the change in the person's reality as a positive predictor of the evolution of the recovery process.^45^ For the participants, in addition to the support from family, professionals and peers, as in previous stages, the importance of support from their social network was highlighted to improve their recovery process. Thus, it is known that there is a significant positive relationship between the size and perceived strength of an individual's social network and certain recovery attitudes.^46^

It is worth noting that, for the participants in our study, the fundamental element for the stages of reconstruction and growth was the importance of the individual's own attitude, taking responsibility for this final part of the process. In fact, no obvious differences were identified in the meaning given by the participants to both reconstruction and growth as a person. This principle was already stated by Anthony in one of the first papers on the concept of recovery which stressed the importance of placing responsibility on the individual and not on professionals.^6^ At this point in the process, establishing routines helped individuals to take responsibility^47^ and recover their functionality.^34^ To do so, they had to face reality with an appropriate attitude. Indeed, a relationship has been found between more optimistic attitudes about the process and better outcomes in the recovery process.^34^ In addition, in these later stages, participants expressed the need to learn about themselves and their problem. There is evidence that confirms that when people with mental health problems have a higher level of knowledge regarding their recovery process, they have more positive attitudes towards recovery.^48^

### 4.1 Strengths and limitations

This study has several limitations as well as certain strengths. These findings have provided valuable information on the perception of the people attended at mental health day hospitals on the concept of recovery and its stages. By adopting a descriptive qualitative approach, this study allowed participants to remain faithful to their account while ensuring transparency of the researchers' interpretations. Difficulties were encountered in recruiting participants due to the pandemic caused by the severe acute respiratory syndrome coronavirus 2 virus, given that it was necessary to close the facility where the interviews were conducted on several occasions throughout the year 2021. The fact that the respondents came from a single centre could limit the variety of the data. The characteristics of the participants could affect the transferability of the results to another setting. Regarding the participants, the profiles obtained are representative of the reality of the day hospital studied.

## 5 CONCLUSION

This study explored the meaning of the recovery process and its stages from the perspective of people attending at adult mental health day hospitals. The results provide new knowledge about the most relevant aspects of the recovery experience of the participants in the recovery process, taking into account the different sequential stages of the process.

The participants described the recovery process as a process based on three pillars; the attitude with which it is faced, its harshness and the effort required to undergo the process. In terms of the stages of the process, in the first stage of the recovery experience, the search for hope is the most important aspect for people with mental health problems. Once hope is restored, it enables people to move on to acceptance and reestablishment of identity as the next stage, and in turn, to search for meaning in life. Preparation was highlighted as fundamental for the search for meaning in life. Finally, in the last stage, it is necessary to assume the responsibility for recovery, where the process is dependent on oneself.

## RELEVANCE FOR CLINICAL PRACTICE

This study has implications for the adequacy of nursing interventions in people who are immersed in a recovery process. The findings clarify the perception that people with mental health problems have of the process and its stages, as well as the elements they perceive as facilitators and limiters. This knowledge at the levels of care could help nurses and health care institutions to align the person‐professional perspectives to adapt and individualise interventions to the stage of the recovery process in which the person is. To apply this knowledge in clinical practice, it would be advisable to perform an initial assessment carried out by nursing professionals, taking into account the perceptions and specific needs of each person to use the gathered information to adapt nursing interventions, ensuring they align with the individual needs and perceptions of each patient on their path to recovery. However, further studies are needed and with the participation of individuals during the overall study process.

## AUTHOR CONTRIBUTIONS

**Ana Ventosa‐Ruiz**: Conceptualisation; investigation; funding acquisition; writing—original draft; methodology; writing—review and editing; formal analysis. **Antonio R. Moreno‐Poyato**: Conceptualisation; investigation; funding acquisition; writing—original draft; methodology; validation; writing—review and editing; supervision; resources. **Teresa Lluch‐Canut**: Supervision; methodology. **Isabel Feria‐Raposo**: Methodology; investigation. **Montserrat Puig‐Llobet**: Methodology; supervision.

## CONFLICT OF INTEREST STATEMENT

The authors declare no conflicts of interest.

## Data Availability

The data that support the findings of this study are available on request from the corresponding author. The data are not publicly available due to privacy or ethical restrictions.
